# Functional implications of orientation maps in primary visual cortex

**DOI:** 10.1038/ncomms13529

**Published:** 2016-11-23

**Authors:** Erin Koch, Jianzhong Jin, Jose M. Alonso, Qasim Zaidi

**Affiliations:** 1 Graduate Center for Vision Research, College of Optometry, State University of New York, 33 West 42nd Street, New York, New York 10036, USA

## Abstract

Stimulus orientation in the primary visual cortex of primates and carnivores is mapped as iso-orientation domains radiating from pinwheel centres, where orientation preferences of neighbouring cells change circularly. Whether this orientation map has a function is currently debated, because many mammals, such as rodents, do not have such maps. Here we show that two fundamental properties of visual cortical responses, contrast saturation and cross-orientation suppression, are stronger within cat iso-orientation domains than at pinwheel centres. These differences develop when excitation (not normalization) from neighbouring oriented neurons is applied to different cortical orientation domains and then balanced by inhibition from un-oriented neurons. The functions of the pinwheel mosaic emerge from these local intra-cortical computations: Narrower tuning, greater cross-orientation suppression and higher contrast gain of iso-orientation cells facilitate extraction of object contours from images, whereas broader tuning, greater linearity and less suppression of pinwheel cells generate selectivity for surface patterns and textures.

## Abstract (web summary)

Stimulus orientation in the primary visual cortex of primates and carnivores is mapped into a geometrical mosaic but the functional implications of these maps remain debated. Here the authors reveal an association between the structure of cortical orientation maps in cats, and the functions of local cortical circuits in processing patterns and contours.

## Graphical abstract

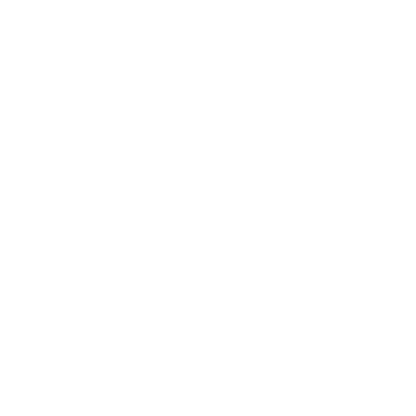

There is a particular satisfaction in neuroscience when anatomical structures can be associated with specific physiological functions. In mammalian brains, this has been accomplished at early stages of the visual pathway but has not been possible for orientation maps in the visual cortex. Here we present experimental and computational results that reveal a significant association between the structure of cortical orientation maps and the functions of local cortical circuits. Hubel and Wiesel^1^ showed that preference for a limited band of orientations is a defining characteristic of neurons in mammalian striate cortex, and that neurons with similar orientation preferences are physically located next to each other in the visual cortex of carnivores and primates. Later studies demonstrated that cortical orientation maps are organized in a mosaic consisting of iso-orientation domains arranged radially around pinwheel centres, where orientation preference changes rapidly along the circular locus^2^,^3^,^4^. Although many models have been proposed to explain how the beautiful geometrical properties of the pinwheel pattern could develop^5^,^6^,^7^,^8^ and why they are remarkably similar across many species, the visual cortices of many mammals including rodents, squirrels and lagomorphs do not have orientation maps^9^,^10^,^11^. Therefore, it remains a mystery whether topographically mapping stimulus orientation is an epi-phenomenon or has significant functional implications^12^,^13^,^14^. Orientation maps are possibly the most established cortical maps and the most tractable to investigate, but the issue of cortical map functions may prove to be much broader as optical imaging and multi-electrode arrays reveal maps in other areas of cortex^15^,^16^.

Nauhaus *et al*.^17^ provided strong evidence that neurons near pinwheel centes are less selective for stimulus orientation than those in iso-orientation domains. Therefore, pinwheel centres could respond better than iso-orientation domains to image patterns made of multiple orientations. Cortical processing of isolated orientations is important for extracting contours and edges^18^,^19^, three-dimensional shape from shading^20^ and the direction of local motion^1^. However, processing multiple orientations simultaneously is essential for more sophisticated visual analyses, such as extracting image patterns^21^, pattern motion^22^,^23^, pattern symmetry^24^, material properties^25^ and three-dimensional shape from texture^26^,^27^,^28^. Even though processing stimuli with multiple orientations is important, the cortical responses to those stimuli are thought to be strongly suppressed by a neuronal mechanism known as cross-orientation suppression. This mechanism reduces the response of a neuron to its preferred orientation when another orientation is present in the stimulus, even if this orientation does not evoke a response^29^. Although cross-orientation suppression was initially thought to originate from intra-cortical inhibition^30^,^31^ and thalamo-cortical synaptic depression^32^, it is now believed to be caused by saturation in the contrast response function of thalamic neurons^33^,^34^. Our results show that the pendulum needs to swing back towards cortical processes to some extent.

The contrast response function of a neuron describes how instantaneous inputs are mapped to outputs and how neuronal responses saturate with contrast^35^. A compressive response function implies that the response to a sum of stimuli will be less than the sum of the responses to the individual stimuli, so response saturation can affect a variety of aspects of visual processing, including spatial frequency selectivity^36^, stimulus salience^37^,^38^ and sensitivity to natural images that contain multiple orientations, both stationary^33^,^34^ and moving^23^. Contrast response saturation is likely to originate at the photoreceptor output^39^ and be effectively transmitted to the thalamus and visual cortex^36^. Pre-cortical contrast saturation thus suppresses cortical responses to stimuli consisting of multiple orientations^33^,^34^ and preventing this from happening is important for pattern perception and requires intra-cortical processing. Our results demonstrate that local discontinuities in the orientation maps (pinwheel centres) allow local cortical circuits to do exactly that.

By performing horizontal penetrations with multi-electrode arrays through cat primary visual cortex, we show that cortical responses from iso-orientation domains and pinwheel centres not only differ in their orientation selectivity but also in their contrast saturation and cross-orientation suppression. We then show that these response differences emerge from two robust computational principles of the visual cortex, excitation from orientation-tuned neighbours and divisive normalization from non-oriented inhibitory interneurons^40^. Based on image processing simulations, we then propose that these differences in contrast saturation and orientation suppression, created by local circuits within the orientation map of primary visual cortex, allow downstream neurons in higher levels of cortex to specialize for detecting either edges and contours of objects, or surface textures and patterns.

## Results

### Electrophysiology

**Figure 1:**
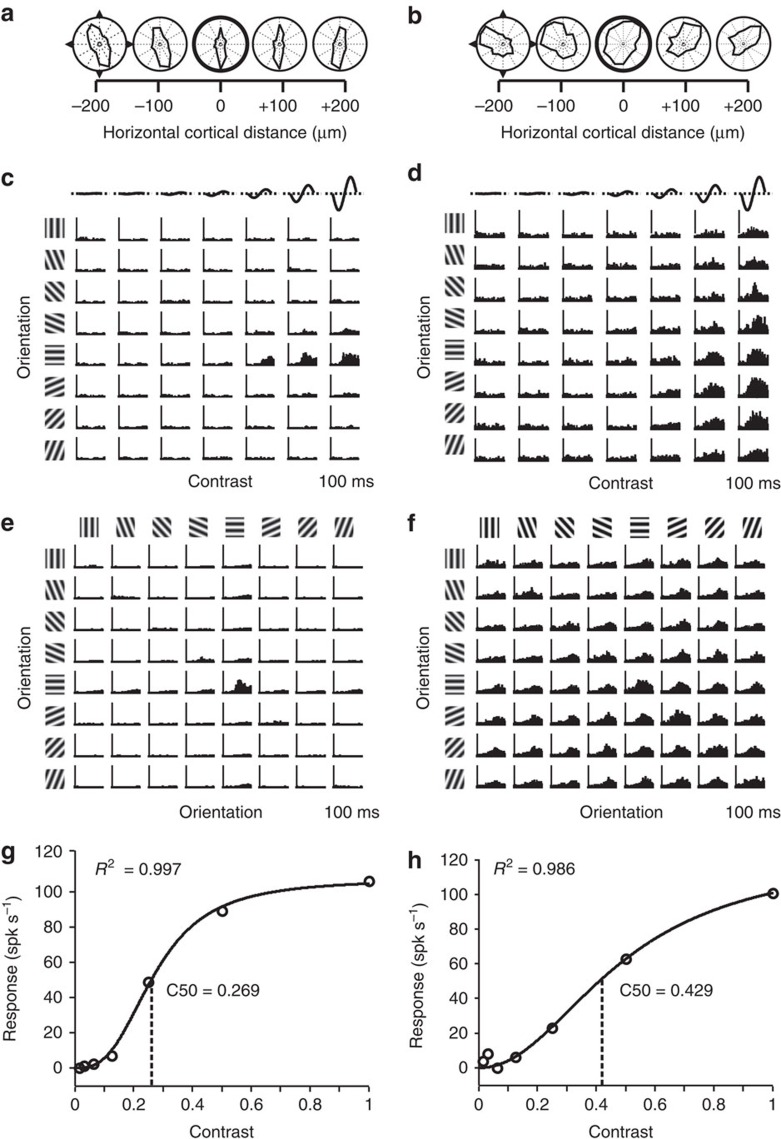
Responses from iso-orientation and pinwheel sites. Recordings from two representative sites using a 32 channel probe (100 μm between recording sites) inserted tangentially into primary visual cortex. Left column: iso-orientation site. Right column: pinwheel site. (**a**,**b**) Polar plots showing the orientation preference for each of the two sites and their nearest two sites on either side, separated by 100 μm. (**c**) Peristimulus time histogram matrix for gratings oriented at 0°, 22.5°, 45°, 67.5°, 90°, 112.5°, 135° and 157.5° (left margin) at contrasts=1/64, 1/32, 1/16, 1/8, 1/4, 1/2 and 1.0. (top margin). This site responds primarily to horizontal orientations. (**d**) Peristimulus time histogram matrix for the same gratings for a broadly tuned site. (**e**,**f**) Peristimulus time histogram matrix of responses to plaids composed of gratings at 50% contrast, oriented as in the top and left margins. (**g**,**h**) Responses to increasing contrast of single gratings of preferred orientation, fit with a Naka–Rushton function. Lower semi-saturation constant (C50) corresponds to greater response compression.

We performed simultaneous recordings from neurons that were horizontally arranged in cat visual cortex using linear arrays of 32 electrodes with 100 μm separation between electrodes. These recordings allowed us to distinguish horizontal tracks passing through iso-orientation domains (Fig. 1a), which showed limited changes in orientation preference and high orientation selectivity, from those running near pinwheel centres (Fig. 1b), which showed rapid changes in orientation preference and low selectivity. The difference in orientation selectivity between iso-orientation domains and pinwheel centres held at different contrasts (Fig. 1c,d) and differences in orientation processing were even more obvious when using grating plaids as stimuli. In the iso-orientation domain (Fig. 1e), cortical responses were driven when the components of the grating plaid had the same preferred orientation (horizontal), but were severely suppressed when the orientation of one of the grating components was different. Conversely, in the pinwheel centre (Fig. 1f), cortical responses were driven by a large variety of grating plaids and multiple orientation combinations. In addition, Naka-Rushton fits of contrast response functions (Fig. 1g,h) showed greater saturation (lower C50 values) in the iso-orientation domain than the pinwheel centre (C50: 0.269 versus 0.429). Next we quantify the population variation of these properties across orientation domains and then show how they arise from local circuit computations.

To quantify the influence of orientation map topography, we calculated the Local Homogeneity Index (LHI) of the horizontal track in which the cortical response was measured, as the rate of change in orientation preference with cortical distance^17^ (see Methods). In agreement with previous studies, we confirmed through visual inspection that sites with lower LHI (closer to 0) are near pinwheel centres and sites with higher LHI (closer to 1) are near iso-orientation domains. Our ranges of LHI estimates are also similar to previously reported values in the cat (multi-unit activity, MUA: 0.1282–0.9670, median=0.7857; single-unit activity, SU: 0.2855–0.9646, median=0.6252).

**Figure 2:**
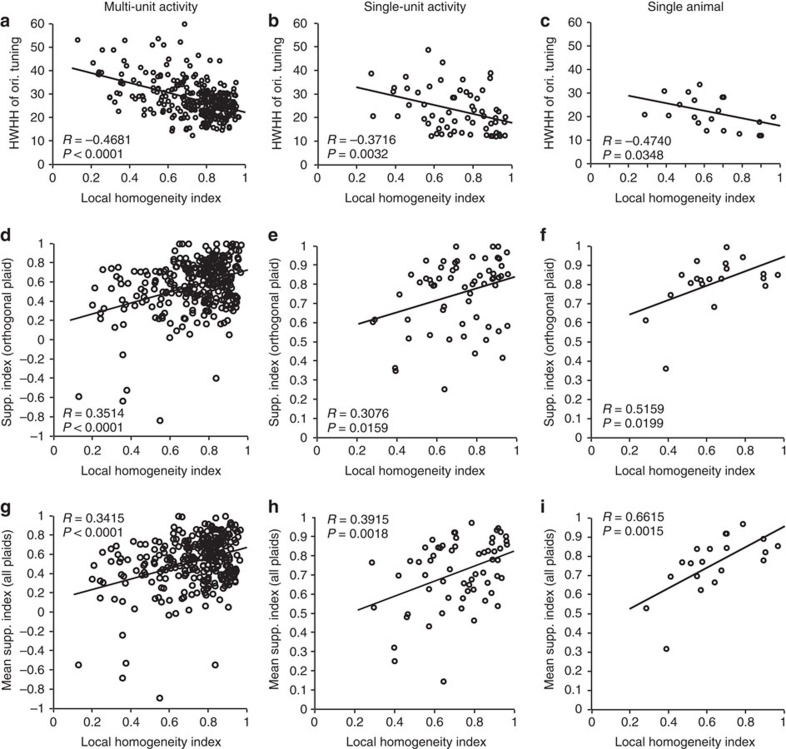
Association between stimulus orientation and cortical orientation topography. Left column: multi-unit activity (*N*=303; 6 cats). Middle column: single neuron activity (*N*=61; 6 cats). Black lines depict best-fitting linear regression. Right column: single neuron activity for one cat (*N*=20). (**a**–**c**) Orientation tuning width versus local orientation homogeneity. (**d**–**f**) SI for the orthogonal plaid (composed of preferred grating and the orthogonal grating) as a function of local orientation homogeneity. (**g**–**i**) Mean SI (average suppression across all plaids containing the preferred orientation) as a function of the local orientation homogeneity.

We first demonstrate the effect of local orientation structure on orientation processing. Consistent with Nauhaus *et al*.^17^, the LHI was negatively correlated with orientation tuning width in our measurements (Fig. 2a–c, MUA: R=−0.4681, *P*<0.0001, *n*=303 (6 cats); SU: *R*=−0.3716, *P*=0.0032, *n*=61 (6 cats); SU single cat: *R*=−0.4740, *P*=0.0348, *n*=20), that is, neurons located in iso-orientation domains with high local homogeneity had narrower orientation tuning than those located in regions with low local homogeneity (putative pinwheel centers). Second, we demonstrate that the processing of multiple orientations also varies systematically across orientation domains. In fact, cross-orientation suppression was often so strong that the response to the plaid was less than the response to just the grating component at the preferred orientation. We calculated a Suppression Index (SI) as 1 minus the response to the plaid divided by the response to the preferred component. Cross-orientation suppression is most often demonstrated with added orientations orthogonal to the preferred orientation and for such plaids (Fig. 2d–f) we found a clear positive relationship between LHI and SI (MUA: *R*=0.3514, *P*<0.0001; SU: *R*=0.3076, *P*=0.0159; SU single cat: *R*=0.5159, *P*=0.0199). In addition, when mean suppression over all the plaids containing the preferred orientation was considered (Fig. 2g–i), we found a similar range to the orthogonal plaid, as was the relation to the LHI (MUA: *R*=0.3415, *P*<0.0001; SU: *R*=0.3915, *P*=0.0018; SU single cat: *R*=0.6615, *P*=0.0015). It is noteworthy that Fig. 2d,g show results from six animals and the results for each animal show the positive correlation we describe (*R*=0.6055, *P*<0.0001; *R*=0.59081, *P*=0.00012; *R*=0.46682, *P*=0.0247; *R*=0.3674, *P*=0.02326; *R*=0.3615, *P*=0.0159; *R*=0.28407, *P*=0.123). Cross-orientation suppression is thus stronger for neurons located in iso-orientation domains than for neurons located closer to pinwheels. These correlations suggest that local orientation neighbourhoods within the cortex do in fact contribute to the processing of multiple orientations, most probably fine-tuning the feed-forward suppression from the thalamus. Parenthetically, we find that there is also a significant negative correlation between half-width at half-height of orientation tuning and the mean SI (MUA: *R*=−0.4086, *P*<0.0001; SUA: *R*=−0.5342, *P*<0.0001), and later discuss the implications for pattern perception. For 6 out of 303 multi-unit sites, the SI is negative, that is, the response to the plaid was larger than the response to the preferred component, but there are no negative indices for the single cells. Five of these multi-units show broad orientation tuning; thus, the responses to plaids may have summed responses from cells with differing orientation preferences.

**Figure 3:**
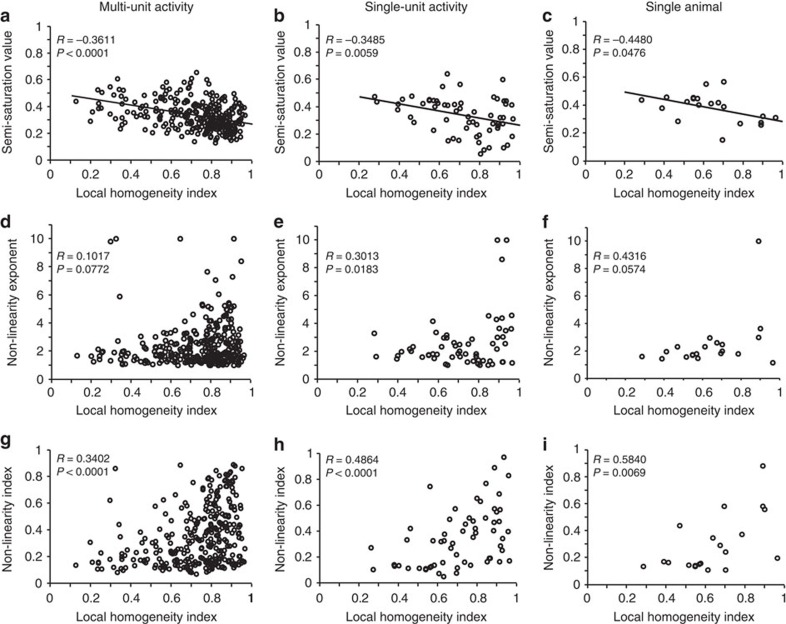
Association between response contrast nonlinearity and cortical orientation topography. Left column: multi-unit activity (*N*=303; 6 cats). Middle column: single neuron activity (*N*=61; 6 cats). Right column: single neuron activity for one cat (*N*=20). (**a**–**c**) Semi-saturation value as a function of local orientation homogeneity. Black lines depict best-fitting linear regression. (**d**–**f**) Exponent of Naka–Rushton fit as a function of local orientation homogeneity. (**g**–**i**) Nonlinearity Index (a value closer to 1 corresponds to a less linear response and a value closer to 0 corresponds to a more linear response) as a function of local orientation homogeneity.

The difference in the contrast response curves across orientation domains (Fig. 1) could also be demonstrated in the population response. The LHI was negatively correlated with the semi-saturation contrast (Fig. 3a–c, MUA: *R*=−0.3611, *P*<0.0001; SU: *R*=−0.3485, *P*=0.0059; SU single cat: *R*=−0.4480, *P*=0.0476), indicating that cortical responses saturate less with increasing contrast near pinwheel centres than in iso-orientation domains. It is noteworthy that when contrast response curves are fit by the Naka-Rushton function, a smaller semi-saturation constant implies earlier saturation. However, the earlier saturation can be altered by a larger exponent that changes the rise of the curve. Interestingly, iso-orientation domains showed smaller semi-saturation constants despite the fact that the exponent of the Naka-Rushton function tended to be higher (Fig. 3d–f, MUA: *R*=0.1017, *P*=0.0772; SU: *R*=0.3013, *P*=0.018; SU single cat: *R*=0.4316, *P*=0.0574). Higher exponents have also been linked to greater stimulus selectivity^35^ and this will turn out to be the case for iso-orientation domains. As no single parameter of the Naka-Rushton curve completely describes the nonlinearity of the contrast response curve, we devised a new nonlinearity index based on departure from linearity from half-maximum response to maximum response, with 0.0 indicating perfect linearity, +1.0 perfect compression and −1.0 perfect expansion (see Methods). Cells in low homogeneity domains were generally close to linear, whereas cells at higher homogeneity domains ranged from almost linear to almost perfectly compressive, yielding a positive relation between response non-linearity and the homogeneity index (Fig. 3g–i, MUA: *R*=0.3402, *P*<0.0001; SU: *R*=0.4864, *P*<0.0001; SU single cat: *R*=0.5840, *P*=0.0069). These results suggest that local computations in cortical orientation neighbourhoods also shape the contrast response function. Importantly, the correlations between the cortical semi-saturation contrast and the mean SI were weak and inconsistent (MUA: *R*=−0.1933, *P*<0.0001; SUA: *R*=0.2089, *P*=0.1060). This result goes against the negative correlation expected between pre-cortical response compression and cross-orientation suppression^33^,^34^. We will later explain the reason for this discrepancy in terms of the cortical computations in our model.

### Excitation–normalization neural model

**Figure 4:**
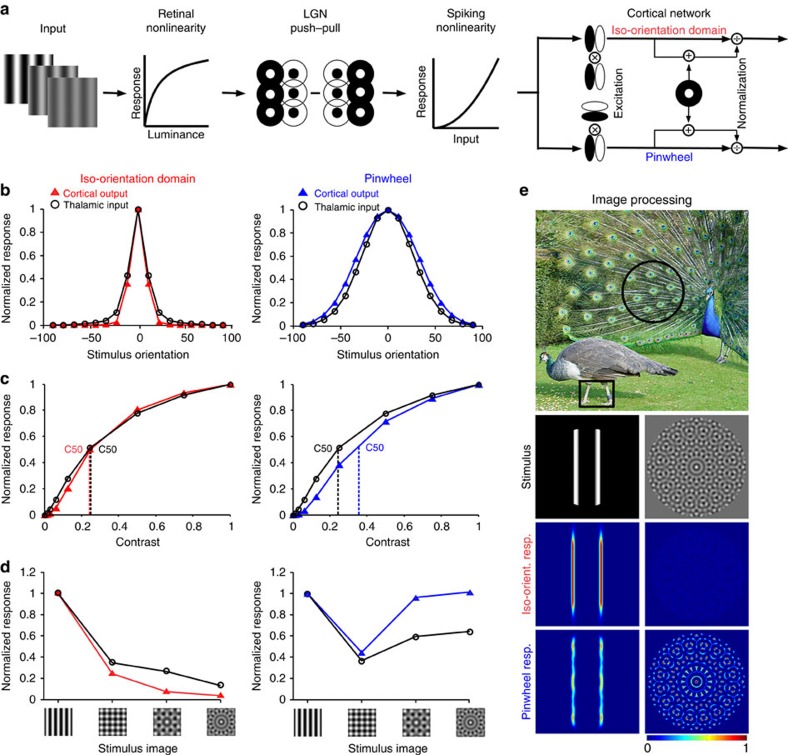
Cortical EN model reproducing differences between iso-orientation domains and pinwheel centres. (**a**) Model schematic: inputs were passed through a photoreceptor compressive nonlinearity. The retinal output was convolved with difference-of-Gaussian LGN receptive fields and combined in a push–pull manner, to create elongated cortical receptive fields that were run through an expansive spiking nonlinearity. Neighbouring V1 neurons had orientation preferences corresponding to iso-orientation and pinwheel domains. The cortical components were excitation from surrounding oriented neurons, followed by divisive self-normalization with the output of a non-oriented inhibitory neuron added to the denominator. (**b**–**d**) Left: predictions for iso-orientation domain cortical output (red triangles) versus thalamic input for narrowly tuned neurons (black circles). Right: predictions for pinwheel domain cortical output (blue triangles) versus thalamic input for broadly tuned neurons (black circles). (**b**) Orientation tuning. (**c**) Contrast response. (**d**) Responses to images of increasing orientation complexity: preferred grating and plaids of 2, 4 and 8 equally spaced orientations including the preferred. (**e**) Top row: a natural image with salient edges and surface patterns (Lu, K. Peacock wooing peahen (Creative Commons Attribution 2.0 License), http://birds.wikia.com/wiki/File:Peacock%2Bwooing%2Bpeahen-2008.jpg, 2003). Second row: images processed through the model: (left) two bars and (right) a rosette pattern. Third row: heat maps showing summed response of eight narrowly tuned model neurons with equally spaced orientation preferences in an iso-orientation domain. Bottom row: heat maps showing summed response of eight broadly tuned model neurons with equally spaced orientation preferences in pinwheel centre.

We used a computational model to investigate the role of the cortical network in shaping the orientation tuning, stimulus selectivity and contrast saturation relayed by thalamo-cortical connections. We set ourselves the task of building the simplest feed-forward model that would simultaneously account for narrower orientation tuning, greater response compression and greater cross-orientation suppression in iso-orientation domains than at pinwheel centres. The retinal, thalamic and cortical components of the model are illustrated in Fig. 4a and the equations are given in Methods. The image was first passed through a photoreceptor compressive nonlinearity^39^ that made the retinal response to a compound stimulus weaker than the sum of the responses to the components. The retinal output was then convolved with thalamic receptive fields modelled as difference of Gaussian functions and the thalamic outputs combined in a push–pull circuit to create elongated cortical receptive fields^41^,^42^. The output of the push–pull stage was then passed through an expansive spiking nonlinearity^43^. These stages predict cross-orientation suppression at the cortical level^33^,^34^, but do not explain how the topography of the orientation map affects the contrast response functions and orientation suppression of the visual cortex.

Using two-photon imaging, Levy *et al*.^44^ found no relationship between the geometry of the dendritic arbour and the local homogeneity in the orientation map, suggesting that all layer 2/3 neurons integrate inputs similarly from all their neighbours. Therefore, we used the same parameters throughout the cortex and applied the same cortical interactions, irrespective of the local arrangement of orientation preference. The first component we used was distance-weighted excitation from surrounding oriented neurons. As cells in iso-orientation domains are surrounded by cells with similar orientation preference, excitation had the effect of differentially increasing the response to the preferred orientation. This, in turn, narrowed the orientation tuning and increased the cross-orientation suppression. In pinwheel domains, excitation from surrounding cells with different orientation preferences differentially increased cell response to non-preferred orientations, thus broadening orientation tuning and decreasing cross-orientation suppression. In addition, the effect of the surround excitation in our model was to reduce compression in the contrast response curves.

Our model assumed the same pre-cortical contrast response across the orientation map; thus, we needed to increase the compression more in iso-orientation domains than pinwheel centres to match the experimental data, while not adversely affecting the other cortical response properties. To achieve this, we used a standard hyperbolic form of self-normalization but replaced the conventional semi-saturation constant by the output of non-oriented inhibitory neurons with severely compressed response functions^45^. As excitation increased responses more for iso-orientation domains than pinwheel centres, a hyperbolic normalization with the same parameters throughout the cortex had the effect of increasing compression more for iso-orientation domains than pinwheel centres, in accord with the physiological results. It is noteworthy that this form of normalization will reduce the amount of cross-orientation suppression when decreasing the cortical semi-saturation constant and thus will reduce the negative correlation between the two values that would be expected if there was no modification of thalamic inputs. We will refer to this model as the excitation–normalization (EN) model.

The combined effects of the two cortical components are illustrated and compared with the input from the thalamus in Fig. 4b–d. The population receptive field of thalamic afferents is less balanced in ON and OFF responses at pinwheels, thus leading to a tendency for broader orientation selectivity than in other cortical domains^46^. The excitatory cortical circuit further narrows the orientation tuning from the thalamic components in the iso-orientation domain as illustrated for a narrowly tuned cortical input in Fig. 4b (left). It also broadens the tuning in the pinwheel neighbourhood as illustrated for a broadly tuned cortical input in Fig. 4b (right). As a consequence, the tuning distribution is narrower for the iso-orientation domain than for the pinwheel centre, in agreement with the experimental results. Moreover, even though the normalization component is identical in the two cortical regions, it yields more saturation in cells within iso-orientation domains than pinwheel centres (Fig. 4c). For completeness, Supplementary Fig. 1 shows the effects of intra-cortical EN circuits on narrowly and broadly tuned neurons within each cortical region (iso-orientation domain and pinwheel).

To predict the EN model's performance in more complex stimulus patterns, we compared the thalamic and cortical outputs generated in response to a sinusoidal grating and plaids made of 2, 4 or 8 gratings with equally spaced orientations including the preferred one. A narrowly tuned cell exhibits cross-orientation suppression just from the thalamic input, as would be expected from the early response compression. The response decreases with the addition of more orientations to the stimulus, because contrast is reduced for the preferred grating to keep the total contrast equal to 1.0 (Fig. 4d left, black). In addition, suppression is further enhanced by the cortical network in iso-orientation domains (Fig. 4d left, red). The broadly tuned cell exhibits cross-orientation suppression that is similar to the narrowly tuned cell, due to the same early response compression. However, the broadly tuned cell responds to the additional oblique gratings; therefore, the responses to four and eight grating plaids increase (Fig. 4d right, black). In addition, the cross-orientation suppression is further reduced by the cortical network in pinwheels, leading to enhanced responses for the complex plaids (Fig. 4d right, blue).

The difference between iso-orientation domains and pinwheel centres is seen clearly by processing patterns and contours through two simulated extra-striate cells, one pooling outputs from eight cells in an iso-orientation domain and the other from eight cells in a pinwheel centre (Fig. 4e). The former responds strongly to the isolated contours and hardly at all to the pattern. The latter gives a weak response to the isolated contours, but recreates the pattern. As a result, extra-striate neurons that pool outputs from iso-orientation domains will provide information about isolated edges and contours, whereas extra-striate neurons that pool outputs from pinwheels will provide information about patterns and textures.

**Figure 5:**
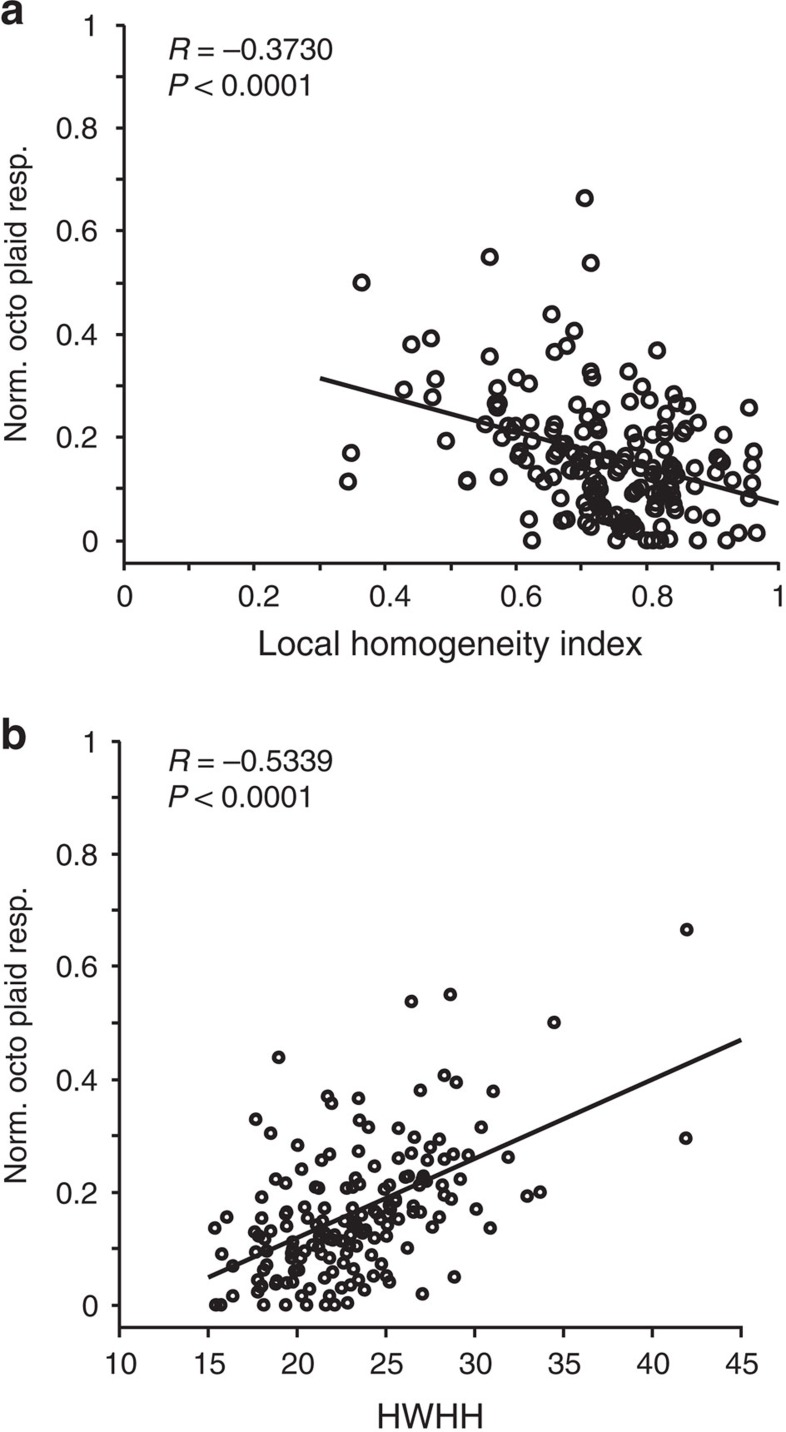
Association between multiple orientation stimulus response versus local orientation topography and orientation tuning width. Stimulus was an octotropic plaid composed by summing eight gratings equally spaced in orientation. Multi-unit activity from three cats (*N*=167). Octotropic plaid response is normalized to the maximum response of the unit. (**a**) Normalized octotropic plaid response as a function of local orientation homogeneity. (**b**) Normalized octotropic plaid response as a function of half-width at half-height of the orientation tuning curve.

After generating these predictions, we measured responses to pinwheel patterns in new experiments (three cats) and compared them with responses to gratings of preferred orientations. We found that responses to octotropic plaids (eight gratings) are significantly higher in pinwheels than in iso-orientation domains (Fig. 5a, MUA: *R*=−0.3730, *P*<0.0001) and relative responses increase with broadness of tuning width (Fig. 5b, MUA: *R*=−0.5339, *P*<0.0001), thus confirming the EN model's predictions.

In summary, our simulations show that applying the same neural computations uniformly across cortex can generate different response properties in pinwheel centres and iso-orientation domains. The excitatory cortical circuit proposed in our model generates cortical compartments that differ in orientation homogeneity and tuning, but also in contrast saturation and response suppression to stimulus patterns. The narrow orientation tuning, high contrast sensitivity, and pronounced cross-orientation suppression of iso-orientation domains seems ideal to detect edges in natural images. Conversely, the broad orientation tuning and limited cross-orientation suppression in pinwheels should allow processing of stimulus patterns made of multiple orientations such as textures.

**Figure 6:**
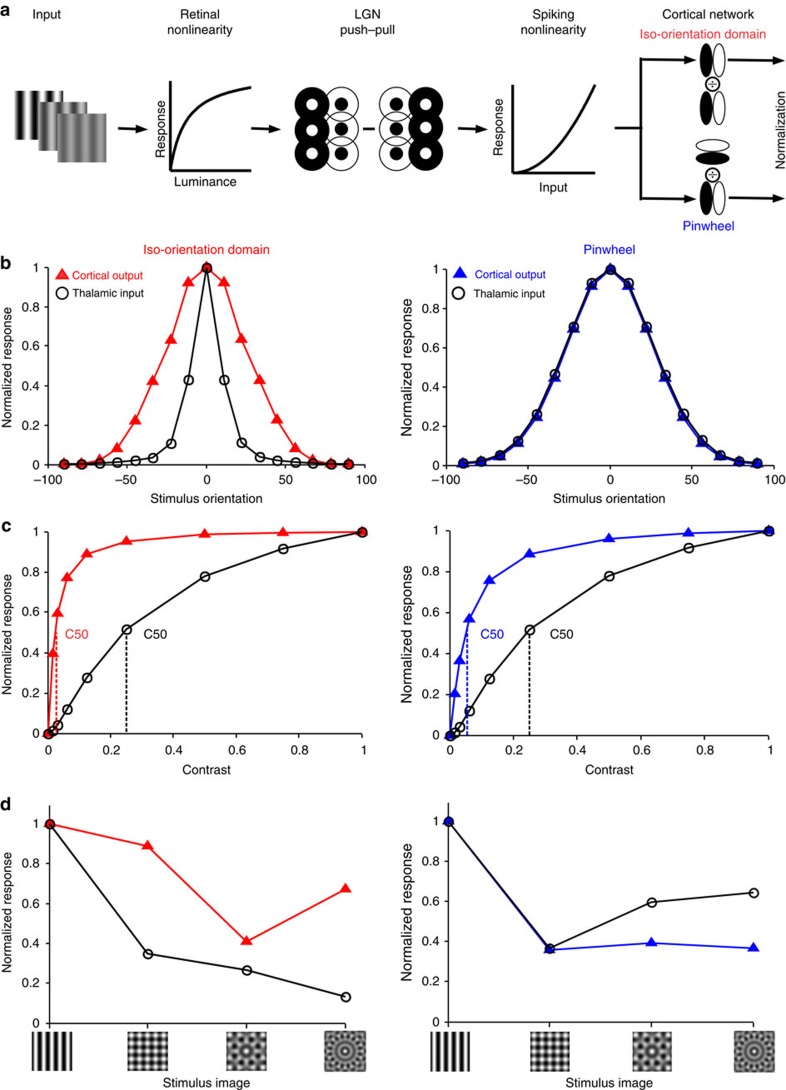
Cortical normalization model predictions for differences between iso-orientation domains and pinwheel centres. (**a**) Model schematic: inputs were passed through a photoreceptor compressive nonlinearity. The retinal output was convolved with difference-of-Gaussian LGN receptive fields and combined in a push–pull manner to create elongated cortical receptive fields that were run through an expansive spiking nonlinearity. Neighbouring V1 neurons had orientation preferences corresponding to iso-orientation and pinwheel domains. The cortical component is normalization from surrounding oriented neurons with a factor added to the denominator, to keep it from going to zero. (**b**–**d**) Left: predictions for iso-orientation domain cortical output (red triangles) versus thalamic input for narrowly tuned neurons (black circles). Right: predictions for pinwheel domain cortical output (blue triangles) versus thalamic input for broadly tuned neurons (black circles). (**b**) Orientation tuning. (**c**) Contrast response. (**d**) Responses to images of increasing orientation complexity: preferred grating and plaids of 2, 4 and 8 equally spaced orientations including the preferred.

Given that divisive normalization seems (theoretically) so omnipresent and accounting for so many cortical response patterns^47^, we present an explicit argument as to why such a normalization is excluded from our model of cortex. We demonstrate that local normalization fails to reproduce the differences in cross-orientation suppression and orientation tuning between iso-orientation domains and pinwheel centres that we empirically measured. To test the effects of local normalization, we used the same pre-cortical components as our model, but replaced the multiplicative surround excitation by divisive normalization from surrounding neurons (Fig. 6a). In iso-orientation domains, divisive normalization from surrounding neurons broadens the tuning curve by reducing responses to preferred orientations (Fig. 6b). In pinwheels, divisive normalization can leave the tuning curve essentially intact (Fig. 6b) or even narrow it by reducing responses to orientations on the flanks, that is, the opposite of the empirical results. Divisive normalization has been commonly used to explain the compression of contrast response curves and here it leads to greater nonlinearity for iso-orientation domains, consistent with the empirical results. However, divisive normalization makes the wrong prediction for processing multi-orientation patterns as shown in Fig. 6d. For completeness, Supplementary Fig. 2 compares the effects of divisive normalization for narrowly and broadly tuned cells in iso-orientation domains and pinwheels.

Divisive normalization could be made consistent with the empirical results by normalizing the responses with distant cells that have orthogonal orientation preferences in iso-orientation domains and the same orientation preferences in pinwheels. This would not only be mathematically *ad hoc* by using different principles for different orientation domains, but would also go against a large body of physiological data. For example, it would predict that the strongest inhibition in most cortical neurons should be evoked by a stimulus orientation orthogonal to the optimal, whereas intracellular recordings have consistently demonstrated that the strongest inhibition is generated by the optimal orientation^48^,^49^,^50^,^51^,^52^. It is noteworthy that there is an essential normalization component to the EN model. This component keeps the excitation within bounds and is responsible for the greater response compression in iso-orientation domains.

**Figure 7:**
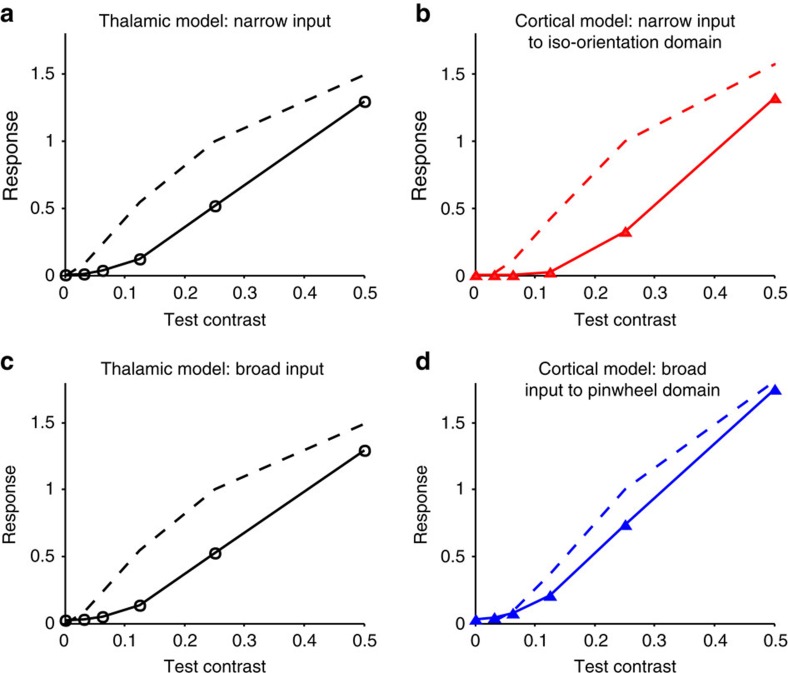
EN model prediction for plaids with varying component contrasts. Predicted responses for (**a**) the thalamic model with narrow input to cortex, (**b**) the EN model with narrow input to an iso-orientation domain, (**c**) the thalamic model with broad input to the cortex and (**d**) the EN model with broad input to a pinwheel centre. Dashed curves are the contrast response curves measured through variation of the contrast of the preferred oriented single grating. Responses are normalized to the 25% contrast model output. The solid lines depict the model predictions for plaids where the preferred orientation is fixed at 25% contrast and the contrast of the component (test) orientation is varied.

Busse *et al*.^53^ generalized measurements of cross-orientation suppression to different relative contrasts of tests and masks. They found summation in cross-orientation suppression when contrasts of the mask and test were similar, but a shift to a winner-take-all rule to mask or test whichever had significantly higher contrast. Busse *et al*.^53^ suggested that the winner-take-all result provided strong support to a surround normalization model expressed in terms of stimulus contrasts. We found that surround normalization was not needed to explain their results. We entered their stimulus contrast values into our model and discovered that even the classical thalamic cross-orientation model predicts summation when contrasts are similar and greater cross-orientation suppression when contrast is higher for one component (Fig. 7). Further, the additional cross-orientation suppression for iso-orientation domains predicted by our model completes the winner-take-all. Busse *et al*.^53^ presented population results for cat visual cortex and, as iso-orientation domain cells dominate in numbers, our model works well as an alternative to divisive normalization in reproducing both the summation and winner-take-all regimes.

## Discussion

Our physiological results demonstrate that cortical neurons in iso-orientation domains not only have narrower orientation tuning, but also generate visual responses that are more sensitive to contrast and more suppressed by non-preferred orientations than cells in pinwheel centres. Neurons near pinwheel centres are more broadly tuned for orientation and generate visual responses that are more linearly related with contrast and less suppressed by patterns made of multiple orientations. The fact that cross-orientation suppression varies across different regions of the orientation map suggests a possible role for the cortical network in shaping neuronal response properties. Using a computational model, we show that applying the same cortical computation homogeneously across primary visual cortex, we can generate outputs that differ in contrast sensitivity, orientation tuning width and cross-orientation suppression, as the cortical orientation gradient varies between iso-orientation domains and pinwheel centres. As variation in these outputs can be used to process different features of natural scenes, our results have broader implications for cortical processing and provide a possible functional role of orientation maps in the visual cortex.

We propose the simplest model that can reproduce the empirical associations that we discovered between cortical topography and neuronal response properties. The most important component is the cortical excitation from surrounding neurons^48^. In iso-orientation domains, excitation has the effect of narrowing the orientation tuning and increasing cross-orientation suppression. In pinwheel domains, excitation broadens orientation tuning and decreases cross-orientation suppression. The effects of orientation topography thus supplement evidence for the amplification of tuned thalamic excitation by cortical circuits^54^,^55^,^56^,^57^.

The EN model needs a normalization component to replicate the physiological differences in contrast saturation between iso-orientation domains and pinwheel centres. Surround normalization is widely proposed as the mechanism for many kinds of cortical effects^47^,^58^; however, we chose not to use it because it had undesired effects in iso-orientation domains and pinwheel centres. As neurons in iso-orientation domains are surrounded by cells with similar orientation preference, surround normalization strongly reduced the response to the preferred orientation, broadening the orientation tuning and weakening cross-orientation suppression. On the other hand, because cells in pinwheel centres have different orientation preferences, the surround normalization narrowed the orientation tuning and strengthened cross-orientation suppression. These effects are just the opposite of what physiological measurements demonstrate. It is worth noting that pinwheel centres constitute a small part of striate cortex. Therefore, our simulations indicate that surround normalization will generally broaden orientation tuning in cortical orientation maps, contrary to what is usually presumed in normalization models.

Making response functions more compressive in iso-orientation domains than pinwheel centres required using a normalization approach that was different from surround normalization. Therefore, we used a standard hyperbolic form of self-normalization, but replaced the conventional semi-saturation constant by the output of non-oriented inhibitory neurons with severely compressed response functions, which are common in the input layers of the visual cortex^40^,^45^,^59^. It is noteworthy that in the absence of intra-cortical inhibition, response redundancy would be greater in iso-orientation domains than pinwheel centres. Therefore, the stronger normalization in iso-orientation domains has the additional benefit of equalizing redundancy across the cortical surface^60^.

The importance of cells that extract contours and edges has been recognized since Hubel and Wiesel^1^; however, extra-striate cells that are selective for specific classes of patterns and textures have only been reported recently^21^,^25^. Our empirical results and model simulations suggest that cells in pinwheel centres provide suitable inputs for pattern selective cells, whereas cells in iso-orientation domains enhance sensitivity to edges. If excitatory connections between similar orientations dominate in the visual cortex of rodents^61^, then these animals could have sharp orientation tuning with high contrast sensitivity and strong cross-orientation suppression even without orientation maps^45^. Therefore, by lacking pinwheels, animals without orientation maps would have limited pattern recognition, but they could still rely on accurate detection of moving edges for survival.

## Methods

### Recording

MUA was recorded from the primary visual cortex of six anaesthetized adult male cats via a 32 channel silicon probes with a 100 μm inter-electrode distance (NeuroNexus Technologies). The impedance of each electrode was ∼1 MΩ. Recorded signals were amplified and filtered by a computer running Plexon (Plexon). Offline software from Plexon was used to sort single units from MUA.

### Animal surgery

Animals were sedated with ketamine (15 mg kg^−1^) and acepromazine (0.2 mg kg^−1^), and anaesthetized with propofol (2 mg kg^−1^). The animals were continuously infused with fluid (2–3 ml kg^−1^ h^−1^) for hydration, vecuronium bromide (0.2 mg kg^−1^ h^−1^) for muscle paralysis, and sufentanil (10–20 ng kg^−1^ h^−1^) and propofol (∼2–5 mg kg^−1^ h^−1^) for anaesthesia through intravenous catheters placed in each hind leg. The eyes were treated with 1% atropine sulfate to dilate the pupil and 2% neosynephrine to relax the nictitating membrane. The refracted eyes were fit with contact lenses with a 3 mm pupil to protect the cornea and focus stimuli on the retina at 57 cm viewing distance. All vital signs were observed and maintained throughout the experiment, including temperature, electrocardiogram, blood pressure, expired CO_2_ and heart rate. Area 17 was exposed by removing the skull and dura overlaying the region. Electrode probes were inserted tangentially with a horizontal angle of less than 5° at the centre of the gyrus. All procedures were performed in accordance to the guidelines of the U.S. Department of Agriculture and approved by the Institutional Animal Care and Use Committee at the State University of New York, College of Optometry.

### Stimuli

We stimulated V1 neurons by monocular presentation of different visual stimuli on a 20-inch CRT monitor (Nokia 445Xpro, Salo). Two sequences of full field stimuli were generated and presented using Matlab routines in the Psychophysics Toolbox. The first set consisted of single sinusoidal gratings at eight equally spaced orientations: 0°, 22.5°, 45°, 67.5°, 90°, 112.5°, 135° and 157.5°. Each stimulus was flashed at four different phases: 0, pi/2, pi and 3pi/2 radians, and at seven different contrasts: 1/64, 1/32, 1/16, 1/8, 1/4, 1/2 and 1. The second set consisted of plaids made by adding pairs of 50% contrast gratings in all possible phase and orientation combinations. Each image was flashed for 100 ms and followed by 100 ms of a blank mid-grey screen. In both sets, the image sequences were randomized and each image was presented 20 times. Multiple blocks of stimuli were run on individual cats.

### Selection of sites

MUA sites used in subsequent analyses had to pass a number of criteria. First, they needed to pass a signal-to-noise ratio (>5), which was calculated as the ratio between mean response of the preferred orientation at full contrast and the s.d. of the least preferred orientation at the lowest contrast. This threshold was determined by careful inspection of peristimulus time histograms that were fit using the Bayesian adaptive regression splines method to get a smooth estimate of firing rate^62^. They were also averaged across all phases and all trials. Response magnitude was quantified as the total number of spikes during stimulus presentation, that is, the area under the Bayesian adaptive regression splines curve. Recording sites were only included in the analyses if it was possible to calculate an LHI for that site. An accurate measure of LHI required at least three recording sites on either side of the reference site to pass the signal-to-noise ratio and contrast response fit requirements (*R*≥0.95). Single units were isolated from sites where it was possible to calculate an LHI (we also required that the contrast response of the single units could be well fit by a Naka Rushton (*R*≥0.95), and that the orientation tuning could be well fit by a Von Mises function (*R*≥0.7)). Peristimulus time histograms for each plaid combination (averaged across all phase combinations and trials) were also fit with a smooth curve using the Bayesian adaptive regression splines method and the magnitude of the response was quantified in the same way as for gratings.

### Contrast response

At each recording site, a contrast response function was fit to responses evoked by single gratings with the preferred orientation at varying contrasts. The classic Naka–Rushton formulation of the hyperbolic ratio was used^63^:


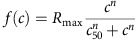


Here, *f* gives the response to a grating of contrast, *c*. *R*_max_ is the maximal response, *c*_50_ is the semi-saturation constant and *n* is the exponent determining the shape of the function. Only recording sites at which the contrast response function could be well fit (*R*≥0.95) were used for subsequent analysis. The parameters displayed on the figures were estimated from the best fitting curves. As *f*(1.0) can be different from *R*_max_, we calculated the maximum response as the fitted function output at 100% contrast and the semi-saturation constant as the contrast value at which the fitted function yields half of the function value at full contrast.

### Orientation tuning width

Orientation tuning curves were fit to the full-contrast single-grating responses at each site with a Von Mises function. We used the half width at half height as a measurement for orientation tuning bandwidth and the stimulus orientation at the peak of the orientation tuning as the orientation preference. Based on the criterion on signal to noise and contrast response fit described above, all multi-units that remained in the analysis were generally well fit by the Von Mises function (mean *R*=0.9208). We also calculated the circular variance as an estimate of orientation tuning width. Results were fairly similar for the two measures.

### Suppression index

A SI was determined for each plaid containing the preferred orientation at each recording site:


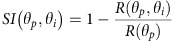


*R*(*θ*_*p*_, *θ*_*i*_) represents the response to a plaid that includes the preferred grating, *θ*_*p*_, and *R*(*θ*_*p*_) represents the response to the preferred grating at 0.5 contrast. This SI highlights the fact that many plaids evoke responses smaller than their preferred component.

### Local homogeneity index

The tangential insertion of the multi-electrode array and inter-electrode distance of 100 μm allows us to estimate the location of each recording site within the orientation map. We used a one-dimensional (1D) version of the Nauhaus *et al*.^17^ LHI, to measure how quickly the orientation preference is changing around each site. The LHI for a given site was calculated by finding the magnitude of a vector addition. Each neighbouring neuron contributes a vector to the sum. The angle of the vector is given by the preferred orientation and the magnitude by a Gaussian weighting of cortical distance from the reference neuron. Explicitly the LHI for a given neuron is:


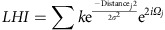


The sum is over all neighbouring neurons. The distance_*j*_ measures how far neuron *j* is from the given neuron in microns; *Ω*_*j*_ is the orientation preference of neuron *j*; *σ* is set to 180 μm (our measurements are spaced by 100 μm); And *k* is a constant equal to the inverse of the theoretically maximum LHI, to ensure that LHI ranges from 0 to a maximum of 1. We use the same spatial Gaussian at all locations and analysed recording sites that were surrounded by other sites that passed our inclusion criteria. Therefore, all locations have the same number of elements contributing to the index.

**Figure 8:**
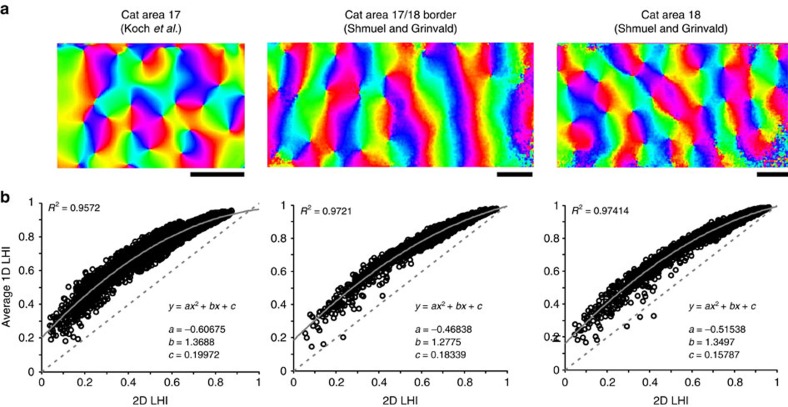
LHI computation simulations. (**a**) Three different orientation maps generated from optical imaging data from our lab (left) and data published by the Grinvald lab (middle and right). Scale bars below maps represent 1 mm in length. (**b**) Plots comparing 2D LHI calculation around 5,000 randomly chosen map locations with the average 1D LHI calculation of 8 radial angles of penetration. Solid grey lines depict the quadratic best fit polynomial. *R*^2^ values are given for this curve and the best-fit coefficients. Dashed grey lines depict the line of unity.

We have checked our 1D estimates of homogeneity against two-dimensional (2D) estimates obtained from two published orientation maps^64^ and our own orientation map. First, we calculated the orientation maps (Fig. 8a). Then, we compared the 2D LHI with the mean of eight 1D LHI measured at equally spaced penetration orientations in multiple locations (Fig. 8b). Finally, we estimated the chance that recordings in pinwheel domains were misclassified as iso-orientation domains in our 1D recordings. We found that the 1D and 2D estimates are strongly correlated and are accurately described by a slightly curved function. Therefore, although the 1D LHI may slightly overestimate homogeneity, no area with 2D LHI of <0.2 will be classified as iso-orientation, as the 1D LHI will at most be 0.2 higher. These simulations show that linear arrays can be reliably used to identify and investigate orientation domains.

### Nonlinearity index

To capture the saturation of the contrast responses curve with a single index, we joined the point of the curve corresponding to half the maximum response to the point of the curve corresponding to the maximum response. We then calculated the area between this line and the corresponding segment of the curve, positive for above the line and negative for below. We then normalized this value by the area between this line and the curve of maximum saturation. The curve of maximum saturation is formed by a vertical line from half the maximum response (half of the function output at 100% contrast) to the horizontal line at the maximum response (the function output at 100% contrast). Hence, the Nonlinearity Index ranged from −1.0 for maximum expansion, to 0.0 for linearity, to 1.0 for maximum saturation.

### Model specification

The luminance value of each pixel of a stimulus *S*(*x*,*y*) is passed through a point-wise Naka–Rushton function to get the photoreceptor output, *P*(*x*,*y*). Here, *L*_50_ is the semi-saturation luminance:


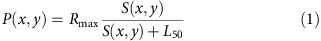


The photoreceptor output *P*(*x*,*y*) is convolved with the receptive fields of ON-Center and OFF-Center thalamic neurons, modelled as difference of Gaussian functions, *D*(*x*,*y*), to obtain the thalamic responses (*T*_on_ and *T*_off_):


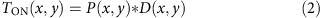



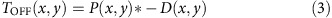



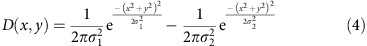


*σ*_1_ is the s.d. of the receptive field centre and *σ*_2_ is the s.d. of the surround (*σ*_1_ is 1/3 of *σ*_2_).

The total thalamic input (*T*_tot_) to a cortical neuron with a preferred vertical orientation is formed by aligning multiple thalamic receptive fields in a vertical row and combining them with a push–pull mechanism:


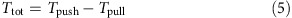



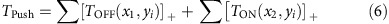



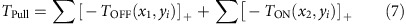


where *x*_1_ and *x*_2_ define the vertical columns over which the *i* thalamic inputs are summed after they are rectified. The cortical orientation tuning was made narrower by increasing the number of thalamic neurons (*i*) and making longer vertical columns. Responses of neurons at other orientations were calculated by rotating the stimulus.

The total thalamic input was then passed through a power-law spiking nonlinearity to get the cortical response, *C*:


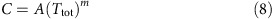


***A*** is a scaling constant adjusted to keep the response of the cortical neuron within a particular range and the exponent *m* is set to 2, which is within the range of measured values.

We added distance-tuned excitation to the model by multiplying the cortical response by a sum of Gaussian weighted responses from orientation-tuned cells in the local cortical neighbourhood, *C*_E_:


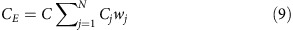



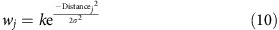


*w*_*j*_ is the weight from each *j*th neuron from the cortical neighbourhood, given by a Gaussian with *σ*=180 μm (as in the LHI calculation) and a constant *k* that is set so that all weights sum to 1.

The response of the cortical cell (*C*_E_) was then self-normalized using a Naka–Rushton function. The normalization included also the output of a non-oriented inhibitory neuron *C*_U_, which acts as the semi-saturation constant in the Naka–Rushton function. The normalized cortical response, *C*_EN_, is given by:


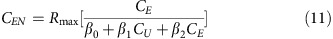


*R*_max_ is the maximum response of the neuron, *C*_U_ is the response of an inhibitory un-oriented cell in the local cortical neighbourhood, *β*_0_ is a small number (set here to 0.01) that keeps the denominator above zero. *β*_1_ and *β*_2_ are weights given to each normalization term. These parameters can be manipulated in simulations, but were left at 1.0 for the predictions in Fig. 4.

It is important to note that the input to the model is exactly the same across the cortex, as are the parameters of local cortical excitation and normalization. The response differences in contrast saturation and orientation suppression arise solely because of the differences in the local distribution of orientation preferences between iso-orientation domains and pinwheel centres (Fig. 4a).

We used a basic form of response normalization for the predictions made in Fig. 6, using thalamic inputs rather than stimulus contrast. The thalamic contribution to the divisive normalization model is identical to that for the EN model. The cortical response from the thalamic afferents was divided by the weighted population response that was previously used for excitation in the EN Model. The response of a cortical neuron for the population normalization model,*C*_N_, is given by:


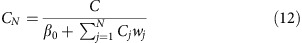


Here, all variables are as before.

### Data availability

Source data available as .mat files and model matlab code is available as .m file upon request.

## Additional information

**How to cite this article:** Koch, E. *et al*. Functional implications of orientation maps in primary visual cortex. *Nat. Commun.* **7,** 13529 doi: 10.1038/ncomms13529 (2016).

**Publisher's note**: Springer Nature remains neutral with regard to jurisdictional claims in published maps and institutional affiliations.

## Supplementary Material

Supplementary Information Supplementary Figures 1 and 2
