# Supplementary material for: Functional implications of orientation maps in primary visual cortex
Source: Nat Commun. 2016 Nov 23;7:13529. doi: 10.1038/ncomms13529 (PMC5122974; doi:10.1038/ncomms13529)
Supplement: Supplementary Information — Supplementary Figures 1 and 2 [file ncomms13529-s1.pdf]

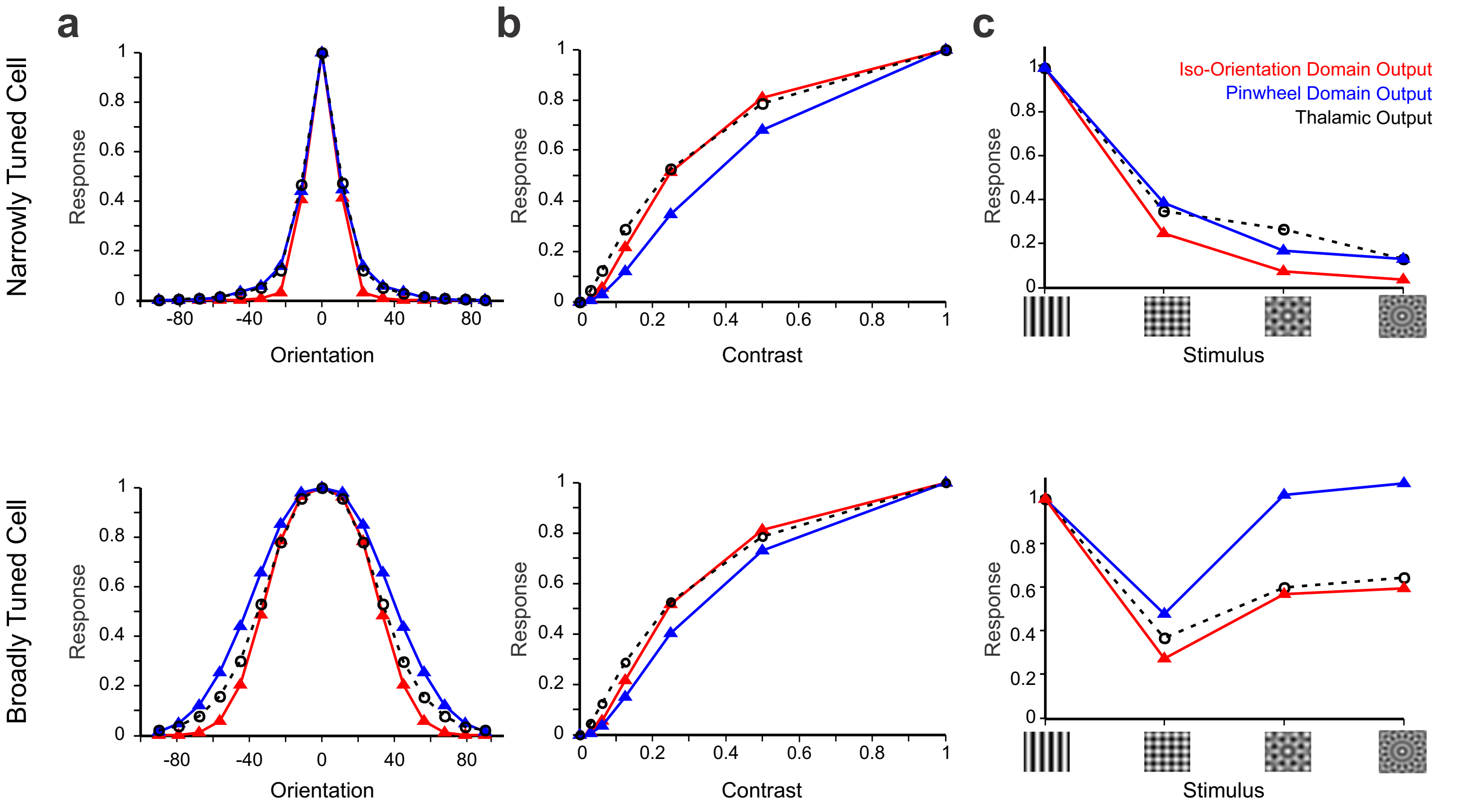

Supplementary Figure 1: Effects of EN circuits for narrowly and broadly tuned cells in pinwheels and iso-orientation domains

a, b, c. Predictions for iso-orientation domain cortical output (red triangles), and pinwheel domain cortical output (blue triangles), versus thalamic input (black circles) for narrowly (Top) and broadly tuned neurons (Bottom). **a.** Orientation tuning **b.** Contrast response. **c.** Responses to images of increasing orientation complexity: preferred grating, and plaids of 2, 4 and 8 equally spaced orientations including the preferred.

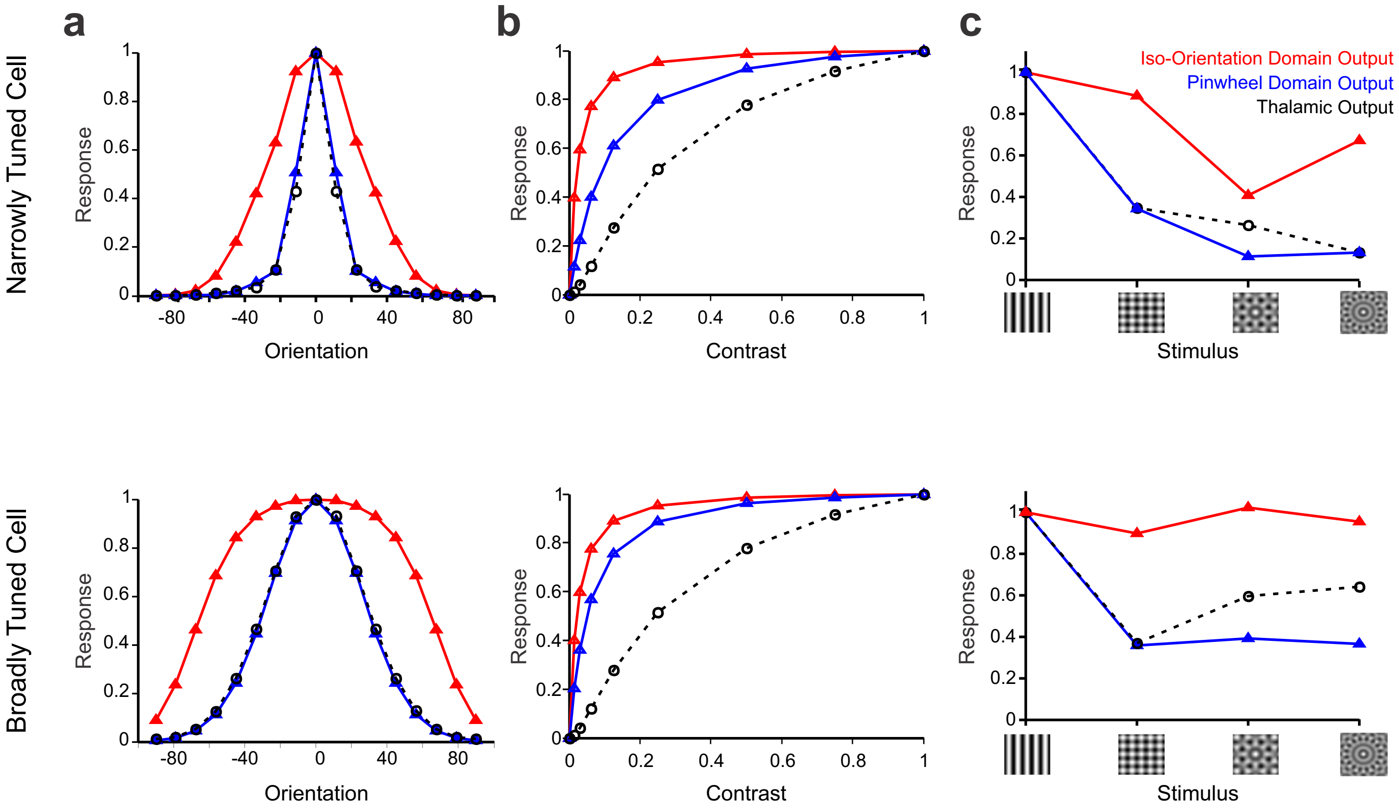

Supplementary Figure 2: Effects of Divisive-Normalization for narrowly and broadly tuned cells in pinwheels and iso-orientation domains

a, b, c. Predictions for iso-orientation domain cortical output (red triangles), and pinwheel domain cortical output (blue triangles), versus thalamic input (black circles) for narrowly (Top) and broadly tuned neurons (Bottom). **a.** Orientation tuning **b.** Contrast response. **c.** Responses to images of increasing orientation complexity: preferred grating, and plaids of 2, 4 and 8 equally spaced orientations including the preferred.
